# Recent advances in targeted mutagenesis to expedite the evolution of biological systems

**DOI:** 10.71150/jm.2501008

**Published:** 2025-03-28

**Authors:** Seungjin Kim, Seungwon Lee, Hyun Gyu Lim

**Affiliations:** Department of Biological Sciences and Bioengineering, Inha University, Incheon 22212, Republic of Korea

**Keywords:** mutations, continuous evolution, targeted mutagenesis, directed evolution, synthetic biology

## Abstract

Evolution has been systematically exploited to engineer biological systems to obtain improved or novel functionalities by selecting beneficial mutations. Recent innovations in continuous targeted mutagenesis within living cells have emerged to generate large sequence diversities without requiring multiple steps. This review comprehensively introduces recent advancements in this field, categorizing them into three approaches depending on methods to create mutations: orthogonal error-prone DNA polymerases, site-specific base editors, and homologous recombination of mutagenic DNA fragments. Combined with high-throughput screening methods, these advances expedited evolution processes with significant reduction of labor and time. These approaches promise broader industrial and research applications, including enzyme improvement, metabolic engineering, and drug resistance studies.

## Introduction

Random mutations and selection toward improved activities have driven the generation of diverse biological systems with unique characteristics via evolution (Molina et al., 2022). Evolution targets include nucleic acids (e.g., DNA and RNA), enzymes, metabolic pathways, cells, etc. Evolution has been adapted to expedite engineering for many biotechnological applications via artificial mutations by physical, chemical, and biological modifications of DNA. However, given evolution mostly relies on genome-wide non-targeted mutations occurring during replication, it requires a large timescale due to a low chance of generating beneficial mutations.

Site-specific mutations are performed to overcome this limitation, especially when a key target or bottleneck is known. Classically, mutations are generated in vitro by error-prone PCR (Cadwell & Joyce., 1992; Yoo et al., 2022) or amplification with degenerated oligonucleotides. Subsequently, these amplicons are cloned into plasmids and introduced into cells. This method can effectively avoid significant challenges in genome-wide mutations, which include the generation of lethal or deleterious mutations and the emergence of cheaters bypassing selection pressure. However, the multiple steps in library generation and low transformation efficiencies for hosts can be significant bottlenecks.

More recently, in vivo targeted mutagenesis strategies have been suggested to enable continuous evolution (Cheng et al., 2021; Molina et al., 2022; Tan et al., 2019). These strategies utilize localized synthesis or incorporation of mutated sequences in cells. Integrated with high-throughput screening, they accelerate evolutionary cycles by enabling the iteration of genetic diversification and the enrichment of desirable strains. These advances are expected to effectively reduce labor, time, and cost while significantly expanding the depth and scale of evolutionary exploration.

This review presents a comprehensive summary of the recent advances in targeted mutagenesis to expedite the evolution of biological systems within living cells. To enhance understanding of the advantages and limitations of each strategy, they are categorized into three main approaches based on their distinct mechanisms for introducing mutations: (i) orthogonal error-prone DNA polymerases, (ii) targeted base editors, and (iii) homology-based recombination. This review also aims to provide insights for selecting the most suitable continuous evolution strategies tailored to specific evolutionary goals. 

## Orthogonal Error-prone DNA Polymerization

Recently, many orthogonal error-prone DNA polymerization systems have been devised for targeted mutagenesis. These efforts require localized erroneous incorporation of bases, which were achieved by using dedicated plasmids, or Cas-based site-specific DNA replacement. Furthermore, mutation rates were increased by lowering the fidelity of DNA polymerase (DNAP). This approach enables all types of mutations, including transitions, transversions, and indels. Transitions refer to the substitution of one purine with another (e.g., A to G) or one pyrimidine with another (e.g., T to C). Transversions are the substitution of a purine with a pyrimidine or the reverse (e.g., A to T or G to C). Indels represent the insertion or deletion of nucleotides. With the ability to generate all these types of mutations, error-prone DNAPs are highly effective for investigating a wide range of genetic possibilities.

### Utilization of the ColE1 origin and error-prone DNA polymerase I

**Fig. 1.**
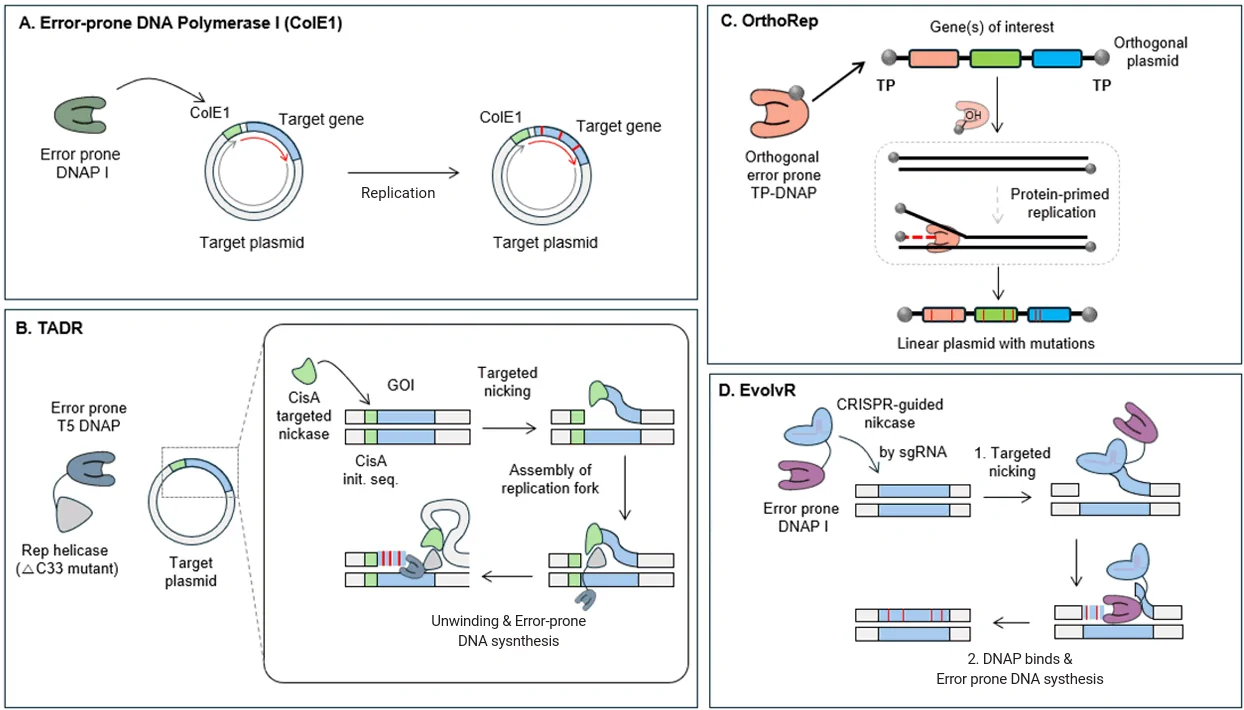
Overview of mechanism in orthogonal error-prone DNA polymerization. (A) Error-prone DNA polymerase I (ep-DNAP I) facilitates targeted mutagenesis near the ColE1 origin by introducing mutations within 3 kb of the replication start site, ensuring localized and controlled mutagenesis. (B) PhiX174 targeting the CisA initiation sequence and creating a nick allows the helicase to recognize the nick and bind to the replication fork, enabling the error-prone T5 DNAP fused with Rep to attach and introduce mutations during DNA replication. (C) OrthoRep is a linear plasmid-based system where an error-prone DNAP associates with a terminal protein (TP) for orthogonal replication, enabling continuous mutagenesis of target genes independently of the host genome. (D) EvolvR utilizes a fusion protein combining nCas9 with an error-prone DNA polymerase. Guided by sgRNAs, nCas9 creates a nick in the DNA, where the error-prone polymerase introduces mutations within a defined window around the nicked site, enabling targeted mutagenesis.

Targeted mutagenesis was achieved by utilizing a pair of the ColE1 plasmid origin and error-prone DNA polymerase I (Pol I, Fig. 1A). Most prokaryotes utilize DNA Polymerase III as the main polymerase for genome replication. In contrast, Pol I plays minor roles such as replacing RNA primers with DNA and repairing DNA (Kornberg & Baker, 1992). However, Pol I is preferentially employed for replicating plasmids with the ColE1 origin. Leveraging this property, a system for targeted mutagenesis in the ColE1-based plasmid was developed with a proofreading-deficient mutant Pol I (D355A and E357A) (Fabret et al., 2000). This approach allowed over 5,700-fold higher mutation rate in the plasmid compared to the genome.[Table t1-jm-2501008]

A fine-tuning of the system was performed to increase the mutation rate in the target plasmid. The utilization of a more error-prone Pol I variant (D424A, I709N, A759R) increased the mutation rate to 8.1×10^-4^ per bp after 15 h of saturated culture (Camps et al., 2003). Additionally, the inactivation of DNA repair systems consisting of MutL and MutS increased the mutation rate by 20–40 times, but it also caused a 300–800 times increase in genomic mutation frequency. Due to a significant increase in the genomic mutation rate, culture conditions were also alternatively optimized, demonstrating that saturated culture conditions were more effective than multiple refreshes in preferentially enhancing plasmid mutation rates over genomic mutation rates.

The developed systems have been applied to facilitate the directed evolution of enzymes to enhance their activity. For example, the activity of α-amylase was enhanced by associating with a growth-based microfluidic droplet screening method (Chen et al., 2024b). Cells with ColE1-origin plasmids harboring an expression cassette for the surface display of α-amylase were grown in media with starch as the sole carbon source. After 25 rounds of screening, a mutant enzyme exhibiting a 1.48-fold improvement in activity was obtained. Another example successfully enhanced the activities of stilbene synthase and *p*-coumarate:CoA ligase, key enzymes in resveratrol biosynthesis (Chen et al., 2024b). Similarly, cells with the ColE1 plasmid designed for the expression of the two enzymes were screened. In this case, the screening was performed by utilizing a resveratrol-responsive biosensor and a fluorescence-activated cell sorter (FACS) screening and cultivation. After 16 rounds of screening, resveratrol production was increased by 1.7-fold. However, the increased titer was attributed not to mutations in the enzyme-coding genes, but to mutations in the plasmid template that led to an increase in plasmid copy number.

### Targeted artificial DNA replisome (TADR) with T5 DNAP fused with Rep helicase

Another strategy employed the replisome of bacteriophage and error-prone T5 DNAP for orthogonal mutagenic DNA replication (Yi et al., 2021). The replisome of the PhiX174 bacteriophage requires only three components: the phage protein nickase CisA, a bacterial Rep helicase, and bacterial DNAP III (Fig. 1B). To construct a plasmid capable of replicating DNA orthogonally to the genome, T5 DNAP was fused with Rep helicase instead of DNAP III. CisA creates a nick in the DNA by recognizing its 30 bp initiation sequence within the plasmid, generating a single-stranded-double-stranded DNA junction. This junction serves as a crucial recruitment site for the Rep helicase. Upon recruitment, the Rep helicase unwinds the DNA strands, facilitating the activity of T5 DNAP, which synthesizes a new strand while displacing the nicked DNA strand, thereby introducing mutations into the target plasmid. This system was 15.3 times more efficient in generating target-specific mutations to ColE1-based tools. Additionally, this system does not rely on the ColE1 origin, expanding its applicability.

The mutation rate was also tuned by applying diverse approaches including structural changes in T5 DNAP, its increased expression, or culture-condition optimization. Specifically, multiple sequence alignments with T7 DNAP and *E. coli* Pol I were performed to identify residues involved in exonuclease activity and substrate recognition, leading to the introduction of D164A, E166A, and A593R mutations to generate an error-prone T5 DNAP. Additionally, designing a stem-loop structure in the 5′ UTR of the fusion protein of Rep and T5 DNAP increased its expression level by 37-fold, leading to a 150-fold increase in the mutation rate. The C-terminal 33 amino acids of Rep, which bind to the innate *E. coli* replisome, were deleted to reduce off-target mutation, thereby improving the selectivity of the system by 16-fold. Cultivation in minimal medium, as opposed to LB medium, resulted in higher targeted mutation rates relative to off-target mutations, with a ratio of 2.37×10^5^. The authors speculate that this improvement may be attributed to a reduced abundance of replisomes in a minimal medium. However, genome-wide off-target mutations still increased by 39.7- to 77.5-fold, possibly due to the overexpression of Rep helicase, which is also involved in genome replication, or the fusion of error-prone T5 DNAP.

The TADR system was exemplarily applied to evolve the *tetA* gene to increase tigecycline resistance, improving the growth rate by 16-fold. In evolutionary testing of a strain carrying a chloramphenicol resistance gene with double early stop codons, reversion mutants with simultaneous mutations in both codons were discovered. This highlights the potential for performance improvements achievable through two synergistic mutations, even in cases where a single mutation alone exhibits no effect.

### Utilization of a self-replicating linear plasmid, OrthoRep

OrthoRep, a continuous mutagenesis system, was developed in yeast using a linear plasmid that replicates independently of the genome (Ravikumar et al., 2014) (Fig. 1C). This plasmid replicates through a distinct mechanism in which terminal proteins (TPs) are covalently linked to the 5′ ends of the linear plasmid DNA. Unlike conventional DNA polymerases that initiate replication using the 3′-OH group of an RNA primer, this system uses the 3′-OH group of a specific amino acid residue within the TP to initiate DNA polymerization by TP-DNAP1. To induce mutations, TP-DNAP variants with single amino acid substitution in the proofreading domain were generated. After screening, the Y427A variant was chosen as it resulted in an increased mutation rate on the plasmid by up to 25-fold (3.5×10^-8^ substitutions per base) compared to the mutation rate of WT TP-DNAP1 (1.39×10^-9^ substitutions per base). As a result, the mutation rate of the plasmid was approximately 350 times higher than that of the genome (10^-10^ substitutions per base).

In a follow-up study, the OrthoRep system was improved to achieve a target mutation rate of 10^-5^ per base per generation, approximately 100,000-fold higher than the mutation rate of the yeast genome, which remained unchanged at 10^-10^ (Ravikumar et al., 2018). A saturation mutagenesis library of 14,000 TP-DNAP1 variants was created by introducing single amino acid substitutions across conserved motifs, which was screened for replication activity and mutation rate changes, yielding 95 promising variants. These variants were then combined and further optimized to enhance the mutation rate, followed by additional exonuclease domain modifications, leading to the final highly mutagenic TP-DNAP1 variant. A global mutation rate of 4.7×10^-6^ per base per generation is known to be lethal to yeast, leading to population collapse. However, OrthoRep overcomes this limitation by restricting mutations to specific target genes, allowing mutation rates far beyond the genome-wide error threshold while keeping cells viable and replicating.

Studies were conducted to enhance the expression level of genes transcribed by OrthoRep for its expanded applicability (Zhong et al., 2018). The linear plasmid is transcribed by a specialized RNA polymerase (RNAP) distinct from those that function in the genome, resulting in OrthoRep transcripts lacking canonical 3′ mRNA polyadenylation. As a result, the transcription and expression levels of plasmid-encoded genes were relatively low, which posed significant limitations, particularly in studies targeting proteins that require high levels of expression. An alternative mechanism was utilized to append 75 A residues, increasing expression levels by up to 10-fold. Furthermore, upstream control regions (UCRs) obtained through evolutionary processes in the previous OrthoRep study (Ravikumar et al. 2018), were hypothesized to enhance expression levels, and experimental validation demonstrated that certain UCRs increased expression levels of target proteins by up to 3-fold. Using various levels of UCRs and poly(A) tails, OrthoRep expression could be controlled across a range of 280-fold.

The OrthoRep system has been utilized to evolve various enzymes, enhancing their resistance, catalytic efficiency, and stability under different conditions. Dihydrofolate reductase (DHFR) was evolved across 90 replicates for resistance to the antimalarial drug pyrimethamine, revealing a fitness landscape that highlighted adaptive trajectories and epistatic interactions (Ravikumar et al., 2018). The system allows all types of mutations but exhibits a strong bias. A:T to G:C transitions account for 73.1% of mutations, G:C to A:T transitions for 21.3%, while other mutation types occur at lower frequencies. Such bias could potentially limit the exploration of all possible sequence variations, constraining the diversity of accessible mutations. *Thermotoga maritima* tryptophan synthase beta-subunit (*Tm*TrpB) was evolved across 10 replicates for 100 generations (Rix et al., 2020). A tryptophan auxotroph was engineered to enable screening, leading to the identification of a variant whose enzyme exhibited a 22-fold improvement in k_cat_/K_M_. A thermophilic enzyme was evolved to exhibit stable expression and activity at 30°C, enabling functionality at a lower temperature. Similarly, THI4, an enzyme inactivated under aerobic conditions, was evolved to enhance its aerotolerance and catalytic efficiency (Gelder et al., 2023). In another study, protocatechuic acid decarboxylase mutants were screened using a transcription factor-based biosensor for muconate and FACS, achieving a 13.7-fold increase in production (Jensen et al., 2021). An automated platform was integrated with OrthoRep to precisely control environmental conditions such as drug concentrations and nutrient levels, dynamically adjusting these factors based on real-time growth data to maintain selective pressure and promote efficient adaptation (Zhong et al., 2020). This approach successfully evolved DHFR for drug resistance, achieving a 30-fold increase in pyrimethamine resistance across 100 generations. Additionally, *Thermotoga maritima* HisA was evolved for mesophilic functionality, enabling stable expression and enzymatic activity at 30°C.

OrthoRep has been expanded beyond yeast to function in bacterial hosts, enabling continuous mutagenesis in a broader range of organisms. OrthoRep, which was previously limited to yeast, was adapted to develop a similar system for bacteria, achieving continuous mutagenesis at a mutation rate 6700 times higher than the genome (Tian et al., 2023). Using this BacORep system, a promoter 36.4 times stronger than the original was evolved, and a 12.3-kb methanol metabolism gene cluster was optimized, resulting in a 7.4-fold increase in methanol consumption. However, this GIL16 DNAP-based system was functional only in *Bacillus thuringiensis* and did not work in *Bacillus subtilis*. The researchers expressed GIL16 DNAP under an inducible promoter, allowing control over copy number from 12 to 80 through the amount of inducer, providing a method to adjust selection stringency or mutation frequency. In addition, an OrthoRep-like system applicable to the commonly used *Escherichia coli* was developed, termed EcORep (Tian et al., 2024). This system employs phage-derived PRD1 DNAP in *E. coli* DH10B, achieving a mutation rate of 9.13×10^-7^ on linear plasmids, which is approximately 1,000 times higher than the genomic mutation rate of 6.4×10^-10^, without affecting the stability of the genome. Using this system, TetA was evolved to increase tigecycline resistance by 150-fold, and GFP was evolved to enhance fluorescence by 1,000-fold.

### CRISPR-based site-specific mutation by error-prone DNAP, EvolvR

Another targeted mutagenesis tool is EvolvR which utilizes error-prone Pol I, fused with nCas9 to create a nick at sgRNA-guided target sites (Halperin et al., 2018) (Fig. 1D). This tool can specifically introduce mutations at target sites by 7,770,000-fold higher than off-target sites. Unlike previous techniques that introduced mutations across the entire plasmid, EvolvR allows targeted mutagenesis in a specific region of the chromosome with a narrower window (10–60 bp). By employing multiple sgRNAs, it is possible to induce simultaneous mutations at various loci. Furthermore, when structural information identifies key residues, the narrow mutation window can be utilized to enhance the quality of the library.

The EvolvR system was improved by optimizing mutation efficiency through parameter adjustments and expanding the mutational window via domain fusion. Mutation efficiency was evaluated by measuring the reversion rate of an early termination codon in GFP, with increased fluorescence indicating higher mutation rates. This enabled the optimization of parameters such as the promoter, polymerase sequence, and culture conditions, achieving a 30-fold increase in mutation rate. Additionally, the mutational window, initially limited to 11 bp, was expanded to 56 bp by fusing the thioredoxin-binding domain of T7 DNAP to the C-terminal of nCas9-PolI3M.

EvolvR has been successfully applied to enzyme engineering to enhance activity and alter substrate specificity, as well as to expand its applicability to yeast. For example, mutations were introduced into ornithine cyclodeaminase, a key enzyme in proline biosynthesis, using three sgRNAs targeting the pocket of the active center. To establish a selection mechanism based on growth rates, common proline codons in an antibiotic resistance gene were replaced with rare codons, reducing translation efficiency and creating a growth bottleneck. This selection pressure enriched cells with enhanced proline production, which carried evolved enzyme variants exhibiting a 2.85-fold increase in its k_cat_ (Long et al., 2020). In another example, the substrate specificity of alditol oxidase (AldO) was altered towards glycerol. Eight sgRNAs were designed at 150 bp intervals to target the entire gene. Cells displaying improved AldO mutants on their surface were screened using a microfluidic platform by associating with the reaction of a glycerol oxidation byproduct (i.e., hydrogen peroxide) and fluorogenic substrate. The final evolved enzyme exhibited increased k_cat_/K_M_ for glycerol by 10.5-fold (Rosenthal et al., 2023). Additionally, the EvolvR system was directly applied to expand the host range to yeast (yEvolvR), achieving a target mutation rate of 1.24×10^-6^, while the global mutation rate increased by 90-fold compared to the wild-type (Tou et al., 2020).

## Base Editor-based Mutagenesis

A method for introducing mutations into DNA using a base editor has been developed (Komor et al., 2016). Originally acting on RNA, cytidine deaminase (CDA) has been adapted to change a base by deamination of cytosine, converting it into uracil. DNAP misreads uracil as thymine, leading to the incorporation of adenine opposite the uracil and resulting in a permanent C to T mutation. Adenosine deaminase (ADA) converts adenosine to inosine, which is recognized as guanosine during replication, thereby inducing A to G mutations. For successful base editing, it is important to recruit the base editor to the target DNA region and to expose it as single-stranded DNA (ssDNA).

A fundamental limitation of base editors is their inability to achieve a wide range of amino acid substitutions, which potentially restricts their utility in protein engineering and directed evolution. CDA alone enables only approximately 9% of all possible amino acid substitutions, and even with the addition of adenine deaminase, the spectrum expands to just around 19%, underscoring their limited capacity to explore the broader sequence space necessary for effective protein evolution (Zimmermann et al., 2023).

### CRISPR-based site-specific base editing

By leveraging the programmable targeting capability of the CRISPR system, CDA was fused with dCas9 for site-specific base editing (Komor et al., 2016). Once dCas9 forms an R-loop complex to expose ssDNA, CDA deaminates cytosine, resulting in a base change to uracil. Since the hybrid of G:U is counteracted by N-glycosylase (UNG), which removes uracil and initiates the base excision repair pathway, multiple approaches were utilized to suppress its activity. They include the utilization of hosts lacking *ung* and the expression of inhibitors of UNG, such as uracil-DNA glycosylase inhibitor (UGI).

### CRISPR-X

**Fig. 2.**
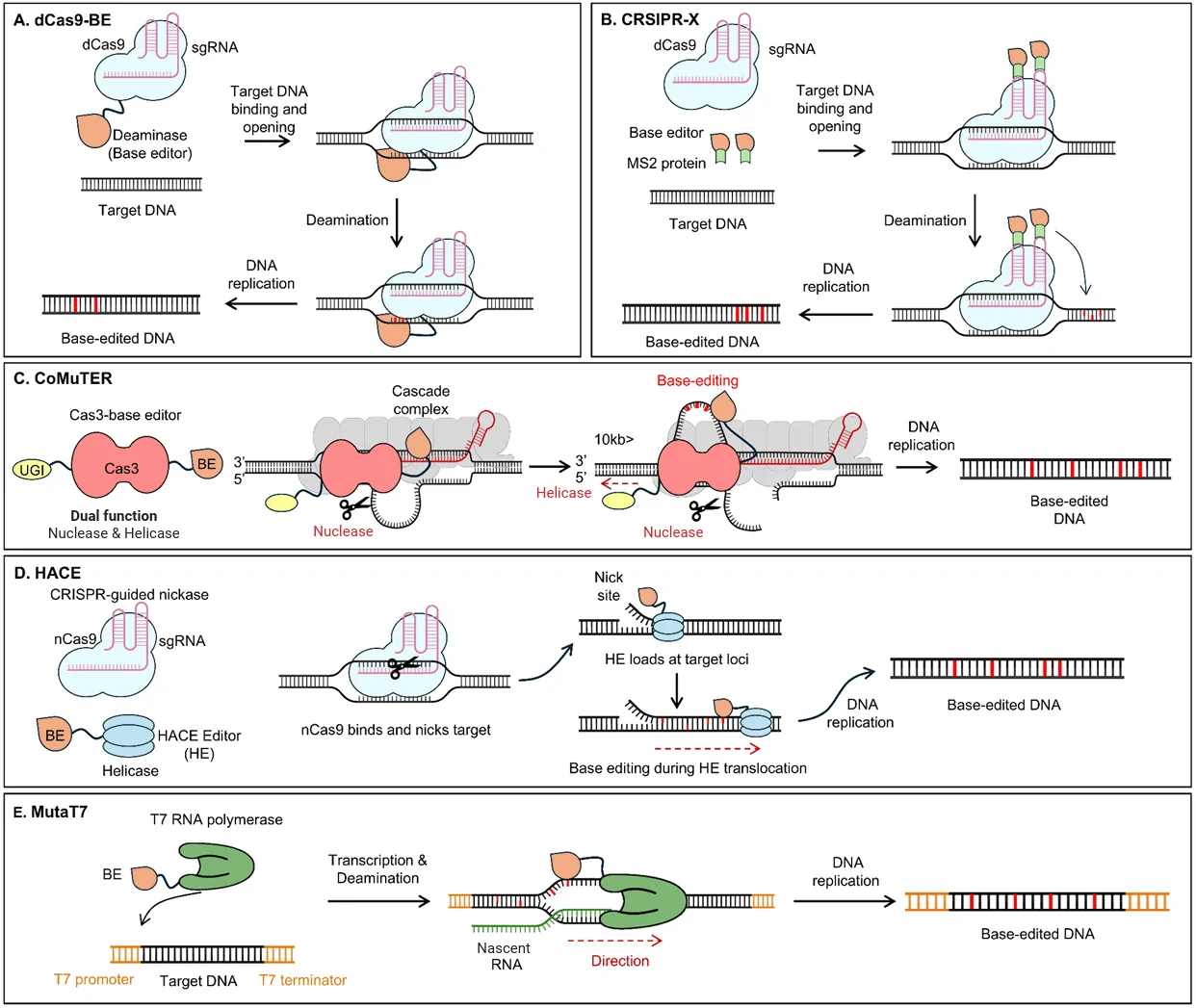
Overview of mechanism in base editor-based mutagenesis. (A) The dCas9-Base editor (BE) is a fusion protein that consists of dCas9 and a deaminase. Guided by sgRNAs, dCas9 exposes single-stranded DNA, allowing the base editor to induce mutations in the target sequence. (B) CRISPR-X employs dCas9, a base editor, and an MS2 fusion protein. The BE-MS2 fusion protein binds to the MS2 region of the sgRNA, translocates to the target site, and induces mutations in the target sequence. (C) CoMuTER utilizes a fusion protein consisting of Cas3, UGI, and a base editor. Helicase and nuclease activities of Cas3 expose single-stranded DNA, enabling the base editor to introduce mutations in a defined region. (D) HACE utilizes a system consisting of nCas9 and a helicase-BE fusion protein. After nCas9 creates a nick in the DNA, the helicase-BE moves to the nicked region, unwinds the DNA, and introduces mutations into the exposed sequence. (E) MutaT7 is a fusion protein of T7 RNA polymerase and a base editor. This system introduces mutations specifically within regions defined by the T7 promoter and T7 terminator during transcription.

CRISPR-X employs dCas9 and MS2-fused CDA to induce targeted mutagenesis (Fig. 2B) (Hess et al., 2016). The MS2 protein naturally binds to a specific RNA hairpin structure from the MS2 bacteriophage genome. To leverage this interaction, sgRNAs were designed to include MS2-binding hairpins, allowing the recruitment of MS2-fused CDA for precise targeting of DNA (Konermann et al., 2015). Once the sgRNA directs dCas9 to the desired DNA region, the R-loop exposes a single-stranded DNA segment. The MS2-fused CDA, recruited via the RNA hairpins on the sgRNA, acts on this exposed single-stranded DNA to efficiently induce mutations. This system allows for targeted mutagenesis within a window of approximately 100 bp, with 77.8% of mutations concentrated in a 20-bp hotspot located between +12 and +32 bp relative to the PAM site. The mutation rate within this hotspot is 5×10^-4^ per bp per generation. Using this system, GFP was mutated to achieve enhanced fluorescence, and resistance mutants of PSMB5, a target of the cancer therapeutic bortezomib, were identified. This technology broadens the applications of base editing, enabling in vivo targeted mutagenesis.

### Confined mutagenesis using CRISPR/Cas3 system, CoMuTER

To expand the editing window of CDA, the Cas3 protein which inherently includes helicase activity, was fused with a CDA to overcome its short editing window, enabling mutations to be extended up to 55 kb (Fig. 2C) (Zimmermann et al., 2023). Cascade complex assembles around a mature crRNA to recognize DNA. After the target site has been recognized, conformational changes in the cascade complex lead to the recruitment of Cas3. Cas3 nicks the non-target strand and loads it into its helicase domain. Subsequent reeling and unwinding of the non-target strand in the 3′-5′ direction generate large stretches of ssDNA, extending the substrate for CDA-mediated mutagenesis.

Using this system, a 9 kb lycopene biosynthesis pathway in *S. cerevisiae* was evolved, successfully identifying an effective S228F mutation in the *crtB* gene for the first time, leading to a 2-fold increase in lycopene production. Similarly, an essential gene, *SEC14* evolved to retain its function while acquiring mutations that confer resistance to nitrophenyl (4-(2-methoxyphenyl)piperazin-1-yl)methanones. Although the intrinsic DNA affinity of CDA increases off-target effects, its site-specific activity achieves a 350-fold higher efficiency than that of the rest of the genome, with an average of 0.3 mutations per kilobase. Given that the expression of Cas3 and CDA has already been demonstrated in plants and mammalian cells, this system is expected to be applicable across various host organisms. However, the nuclease activity of Cas3 occasionally results in deletion events, which could hinder protein evolution.

### Helicase-assisted continuous editing, HACE

To achieve a larger editing window of up to 1,000 bp, an nCas9-helicase fusion was employed as an alternative to Cas3, providing a nuclease-free system that prevents unintended deletions (Fig. 2D) (Chen et al., 2024a). This strategy, called HACE, was developed for continuous targeted mutagenesis on the mammalian genome. It identified variants in mitogen-activated protein kinase kinase 1 that confer resistance to inhibitors. It also identified mutations in splicing factor 3B subunit 1 that are responsible for disrupting canonical splicing. These findings underscore the potential of HACE to uncover how specific mutations drive missplicing, advancing the understanding of splicing biology and informing the development of diagnostic tools and therapeutic strategies for splicing-related diseases. Furthermore, HACE introduced functional artificial variants in the enhancer regions of CD69, identifying specific bases and motifs crucial for the binding of RUNX transcription factors and their regulatory influence on CD69 expression.

### RNAP-mediated site-specific base editing, MutaT7

The MutaT7 system utilizes a fusion protein of a deaminase and T7 RNAP to induce mutations via base editing mainly on the non-template strand exposed during transcription (Fig. 2E) (Moore et al., 2018). The template strand remains hydrogen-bonded with the newly synthesized RNA, resulting in a relatively lower mutation frequency. While the CRISPR-based tools allow mutations in a small window, this system can introduce mutations between the T7 promoter and a terminator, which can be over tens of kilobases. Initial MutaT7 achieved a mutation rate of 6.7 × 10^-6^ mutations per base replicated in *E. coli*, with most mutations occurring on the sense strand, leading to 90% C to T mutations and 10% G to A mutations. The G to A mutations are complementary outcomes resulting from C to T mutations on the opposite strand.

Subsequent optimizations have significantly improved the efficiency and versatility of the MutaT7 system. Modifications to the deaminase enzyme led to the development of eMutaT7^C to T^, which increased the mutation rate to 9.4×10^-5^ per bp per generation (Park & Kim, 2021). To efficiently generate G to A mutations alongside C to T mutations, a dual promoter/terminator approach was implemented, wherein a second pair of T7 promoters and terminators transcribed the target gene in the reverse direction. To reduce the frequent readthrough beyond the T7 terminator that causes off-target mutations, T7-DIVA utilized dCas9 to more precisely control the termination region, achieving a 6.67×10^-5^ mutations per base replicated in *E. coli* (Álvarez et al., 2020). This approach enables precise control over the edited DNA window within a gene, allowing mutations to be confined to specific regions without disrupting the coding sequence.

Expanding the mutation spectrum and reducing strand bias have been key goals in advancing MutaT7 technology. A modified MutaT7 system incorporating the recently developed ADA for base editing was introduced (Mengiste et al., 2023). ADA demonstrated a higher mutation rate with TadA8e, an optimized variant of TadA.10 developed through PACE. Compared to CDA, which exhibits a strong preference for the sense strand, ADA demonstrates a more balanced distribution of A to G mutations, with 45% occurring on the antisense strand. Subsequently, a MutaT7 system was developed that simultaneously utilizes CDA and ADA to introduce all possible transition mutations (Seo et al., 2023). By adjusting the expression levels of both deaminases and UGI, the system achieved a balanced mutation ratio of 50% A:T to G:C and 50% C:G to T:A, with an overall mutation rate of 2.15×10^-5^ per base replicated. There is a need for optimization processes, as well as a concern about the risk of homologous recombination, which arises because the two fusion proteins share a long common region, T7 RNAP. To address these issues, a single deaminase-based MutaT7^GDE^, which enables one enzyme to perform both the distinct activities of CDA and ADA, was developed, establishing a high-error-rate system with 10^-4^ mutations per base replicated in *E. coli* (Mengiste et al., 2024). The original MutaT7 showed significant variation in C to T mutation efficiency depending on the +1 and -1 flanking sequences, but the new system reduces sequence context preference, lowering such dependency and enabling higher mutation rates across a broader range of sequences. C to T mutations were 3.31 times more frequent on the sense strand, and A to G mutations were 1.45 times more frequent on the sense strand. This represents a significant reduction in strand bias compared to the original system. If this bias can be sufficiently reduced, the system may no longer require placing T7 promoters and terminators at both ends of the target DNA. Recently, MutaT7 systems have been successfully extended to a diverse range of hosts, including yeast (called TRIDENT) (Cravens et al., 2021), plants (Butt et al., 2022), human cells (called TRACE) (Chen et al., 2020).

## Homologous Recombination-based DNA Mutagenesis

Homologous recombination enables precise mutagenesis by integrating mutagenic DNA fragments into the target regions through sequence homology. Since this approach utilizes homologous recombination, it is not necessary to pre-edit the target regions, thereby enabling greater flexibility and applicability.

The use of single-stranded DNA (ssDNA) as a template offers significant advantages, such as requiring shorter homology arms, enhancing recombination efficiency, and reducing off-target integration rates (Liu et al., 2024). The ssDNA anneals to the lagging strand template at the replication fork, a step facilitated by the phage λ-Red ssDNA-binding protein. Strategies are required to generate ssDNA and facilitate recombination, ensuring the continuous introduction of mutations.

### Addition of mutant oligonucleotides for multiplex automated genome evolution, MAGE

**Fig. 3.**
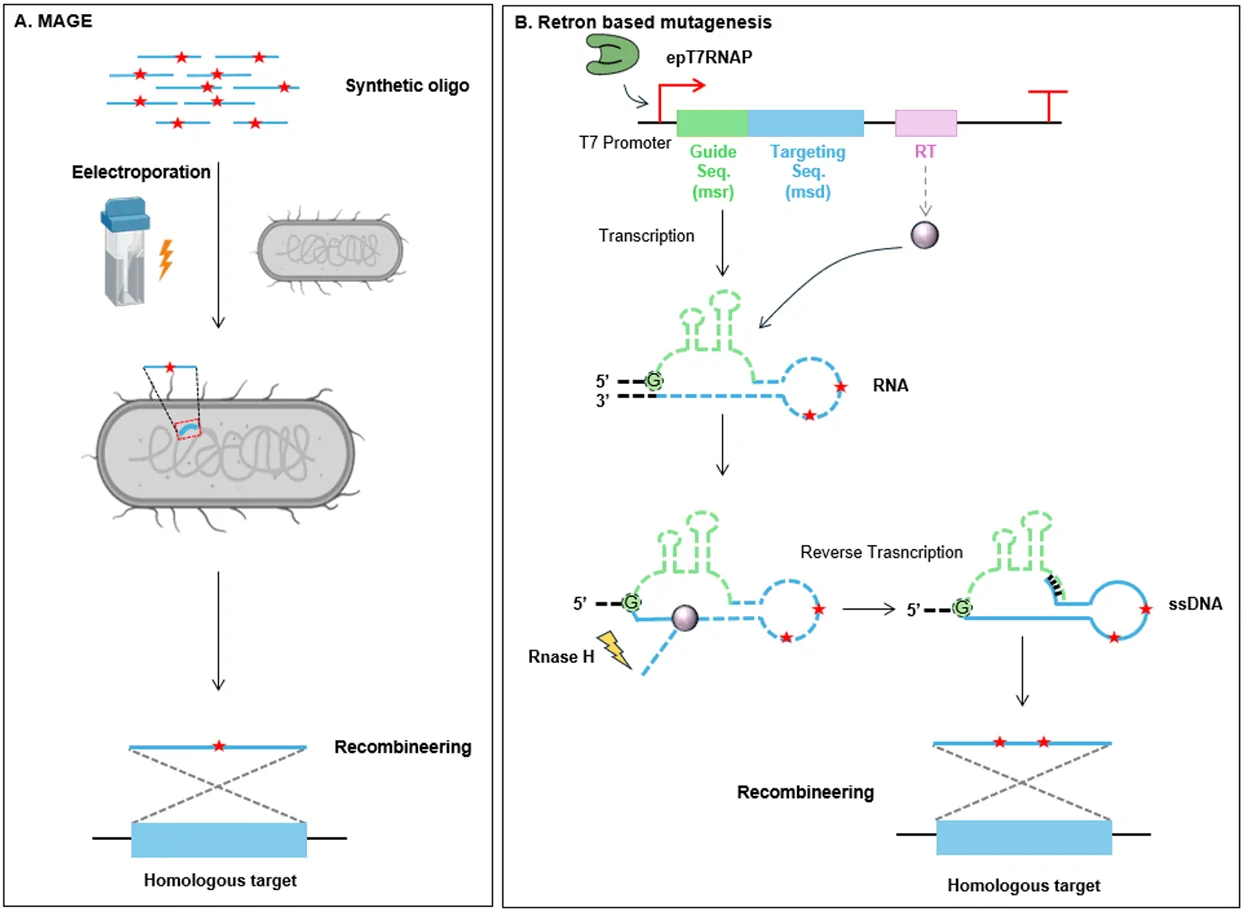
Overview of mechanisms in homologous recombination-based mutagenesis (A) Multiplex automated genome evolution (MAGE) automates the iterative introduction of externally synthesized single-stranded DNA (ssDNA) libraries into cells via electroporation. These ssDNA oligonucleotides recombine with homologous genomic regions during replication, enabling targeted mutagenesis across multiple loci. (B) Retron-based mutagenesis utilizes a retron cassette to produce mutagenic ssDNA in vivo using error-prone T7 RNAP. The resulting ssDNA integrates into homologous genomic regions, facilitating targeted mutagenesis.

MAGE iteratively transforms a library of synthesized single-stranded oligonucleotides via electroporation into *E. coli* (Wang et al., 2009). These ssDNA oligos, of roughly 90 bp, are designed with homologous arms targeting multiple specific genomic loci and interspersed with desired mutations such as substitutions, insertions, or deletions (Fig. 3A).

To improve efficiency, mutation repair systems were inactivated or proteins facilitating homologous recombination were expressed in the host. Specifically, in *E. coli*, *mutS* was removed to inactivate the mismatch repair (MMR) system, and involved in λ-Red recombination (i.e., Exo, Beta, and Gam) were expressed to promote recombination efficiency. When this approach was applied to *Saccharomyces cerevisiae*, the host MMR system (*mlh1*) and overexpressing homologous recombination factors (Rad51 and Rad54) were performed since this approach was not effective in yeast (Farzadfard et al., 2021; Lim et al., 2020; Wannier et al., 2020). Oligo-mediated genome engineering achieved recombination efficiencies ranging from 0.2% to 2.0% in three separate *S. cerevisiae* haploid strains of different lineages (VL6-48, CEN.PK113-7D, and VTT C-68059) (DiCarlo et al., 2013). Further improvements have been demonstrated in eukaryotic MAGE (eMAGE) for yeast (Barbieri et al., 2017), which enhances integration efficiency of oligonucleotide by combining MMR inactivation with strategies like slowing DNA replication using hydroxyurea to facilitate oligonucleotide annealing and employing co-selection methods. pORTMAGE mitigated the increase in background mutations caused by the inactivation of the host MMR system through the implementation of transient MMR inactivation strategy (Nyerges et al., 2016).

As an example, this MAGE strategy was applied to optimize a lycopene biosynthesis pathway (Wang et al., 2009). A total 24 degenerate oligonucleotides were generated to diversify ribosome binding site (RBS) sequences for 20 genes within the pathway and inactivation oligonucleotides for four enzymes in competing pathways. After 35 MAGE cycles, this approach generated 15 billion genetic variants, with the best variant showing a fivefold increase in the production of the lycopene.

While MAGE provides an effective method for generating genetic diversity, its dependence on exogenously synthesized oligonucleotides and automated systems imposes significant constraints. MAGE can be considered an in vitro mutagenesis technique due to the exogenous synthesis of oligonucleotides, the combinatorial diversity it generates occurs within the cells, allowing it to be regarded as limited in vivo continuous evolution. It enables the accumulation of diverse genetic variants through iterative recombination cycles, creating substantial combinatorial libraries of multi-target mutations. However, MAGE faces significant limitations, including the reliance on exogenously synthesized ssDNA, which restricts genetic diversity to the initially designed sequences. MAGE utilizes robotic systems to automate iterative cycles of mutation introduction, giving the appearance of a continuous process from the perspective of the operator. While this approach significantly minimizes manual intervention, its dependence on specialized equipment and automation infrastructure limits its accessibility for many laboratories.

### Retron-based mutagenesis

To enable efficient targeted hypermutation and broaden the scope of continuous evolution applications, a novel strategy utilizing reverse transcription for in vivo ssDNA library generation was developed (Fig. 3B). Retrons are naturally occurring genetic editing systems found in diverse bacterial species (Lampson et al., 2005). These systems generate compact complexes containing reverse-transcribed DNA, which can modify homologous genomic regions. A retron comprises four key components: a specialized reverse transcriptase (RT), an RT guide sequence, known as “msr”, a DNA targeting sequence designated as “msd”, and self-complementary regions. After transcription, the guide and targeting sequences fold into a conserved hairpin structure through complementary regions, serving as a template for reverse transcription. The RT initiates DNA synthesis at the 2′ hydroxyl group of conserved guanosine in the msr and reverse transcribes the msd. Once reverse transcription is complete, cellular RNaseH degrades most RNA of the targeting sequence, leaving a 5–8 base section near the end hybridized to its complementary DNA strand. This hybrid of ssDNA covalently linked to RNA aligns with homologous genomic sequences and drives mutagenesis through homologous recombination. Compared with the introduction of chemically synthesized ssDNA in vitro, the generation of ssDNA in vivo offers several advantages. Retrons synthesize multicopy ssDNA donors in vivo, eliminating the need for external synthesis and delivery while significantly enhancing editing efficiency by supplying abundant ssDNA templates.

To improve the efficiency and applicability of retron-mediated genome editing, various optimizations have been introduced. Using an error-prone RNAP to transcribe the retron cassette, targeted mutations were introduced into the target region (Simon et al., 2018). About 75 bp target homology arm sequence was used to integrate into the target region. Mutation rates were observed to be 190 times higher than background cellular mutation rates. To improve the efficiency of retron-mediated genome editing, optimization efforts were implemented. These included enhancing the T7 RNAP promoter, deleting exonuclease genes such as *exoX* and MMR components like *mutS*, and co-expressing the lambda phage protein Beta. These modifications collectively improved retron-mediated genome editing efficiency by 78-fold and extended the editable sequence length to a maximum of 31 bp.

Expanding retron-mediated genome engineering, a follow-up study called REGES was developed to improve recombination efficiency and multiplex editing capabilities. By assembling an array of retron cassettes, REGES demonstrated recombination at four multi-target loci with a high efficiency of 25% (Liu et al., 2023). Through various optimizations including the deletion of exonucleases such as *xonA* and *recJ*, recombination efficiency was enhanced, enabling successful recombination of sequences up to 300 bp. Additionally, DNA mutations were induced using MutaT7 with approximately 10-fold higher efficiency compared to controls, and RNA mutations were introduced by error-prone T7 RNAP at a rate 50 times higher. Combined with DNA and RNA mutations resulted in an overall mutation rate 1000 times greater than the control. In this study, continuous mutagenesis was primarily applied to inactivate *sacB*, but the improved multiplex editing efficiency through these optimizations suggests broader applications in the future.

Retron-based systems provide advantages over CRISPR/Cas for efficient multiplex genome editing. Until now, multiple targets have primarily been edited using CRISPR/Cas systems with multiple sgRNAs. Homologous recombination-based systems allow multiplex editing in regions lacking PAM sequences. Additionally, sgRNA-based systems, characterized by their reliance on 20 bp target sequences and highly sensitive seed regions, are prone to losing targeting efficiency during continuous mutagenesis, potentially halting the evolutionary process. In contrast, retron-based systems, leveraging approximately 90 bp homology arms, exhibit greater robustness and enable more sustained mutagenesis. Furthermore, retron systems impose less cellular burden due to the absence of Cas9 expression and avoid interference with gene expression caused by dCas9 binding to DNA.

However, retron-based approaches also have key limitations that must be addressed to expand their applicability. The main disadvantages of this strategy are the need for host modifications, such as deleting *sbcB*, *recJ*, *exoX*, *xonA*, which encode proteins that degrade ssDNA, and *mutS*, which encodes a protein responsible for DNA repair during homologous recombination. These requirements confine the strategy to well-characterized strains with established genome engineering tools, limiting its applicability to less-studied hosts. Therefore, further research is required to optimize the retron system, allowing efficient editing to be achieved through plasmid introduction alone, without the need for extensive host genome engineering. Additionally, the current strategy used in bacteria, REGES, has not yet been demonstrated in eukaryotic systems such as yeast, which restricts its broader applicability. 

## Conclusion

The evolution of targeted mutagenesis technologies has revolutionized the ability to engineer biomolecules and biological systems. Orthogonal DNA polymerases, base editors, and homologous recombination-based systems each contribute distinct capabilities, from enhanced precision and reduced off-target effects to robust multiplex editing and continuous mutagenesis. Despite these advancements, enhancing target mutation rates and reducing off-target effects remain fundamental challenges for improving the efficiency of these systems. Beyond these improvements, expanding mutation windows, minimizing sequence context preferences, and reducing mutation biases through advanced base editor development can ensure a more balanced exploration of mutation types, enabling the efficient discovery of beneficial mutations across a broader genetic landscape. Additionally, the reliance on host genome modifications and limited applicability to diverse host systems present significant barriers. Reducing the limitations associated with host systems and minimizing the need for genome modifications through system optimization can extend the applicability of these technologies to novel, specialized non-model organisms, enabling broader industrial applications.

**Fig. 4.**
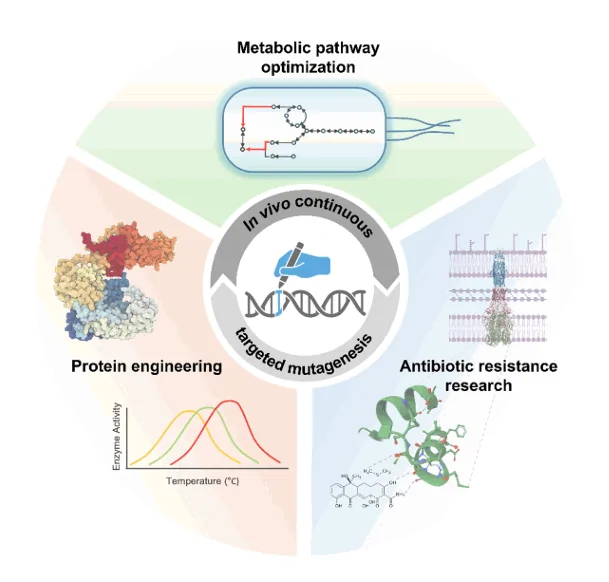
Applications of in vivo continuous targeted mutagenesis. Schematic representation of the applications of in vivo continuous targeted mutagenesis, including metabolic pathway optimization, protein engineering, and antibiotic resistance research. These applications leverage high-throughput screening to drive advances in synthetic biology and biotechnology.

Each method carries unique strengths and limitations, requiring a tailored strategy to select the most appropriate approach, whether it involves orthogonal DNA polymerases, base editors, or homologous recombination-based systems, depending on the specific application. The strengths and limitations of each technology should be strategically utilized to achieve the most effective outcomes, tailored to the specific evolutionary targets and research objectives. The integration of these systems with high-throughput screening and synthetic biology tools promises to accelerate evolutionary exploration, paving the way for breakthroughs across various domains. Beyond current applications such as antibiotic resistance design, these technologies hold immense potential for more complex challenges, including protein engineering, customized strain development, and metabolic pathway optimization (Fig. 4). By enabling precise and efficient mutagenesis, they can significantly enhance the creation of novel biomolecules, streamline the development of tailored biological systems, and advance industrial biotechnology. This transformative potential underscores their capacity to drive substantial progress in both academic research and industrial innovation.

## Figures and Tables

**Table 1. t1-jm-2501008:** Comparison of in vivo continuous targeted mutagenesis methods

In vivo Targeted mutagenesis							
Category	Technology	Number of off-targets	Mutational window (bp)	Host cells	Induced mutation types	Mutation rate	Ref
Orthogonal error-prone DNA polymerization	ColE1/Pol I	High	~4,000	*E. coli*	Transition, Transversion, Indel	8.1×10^-4^	Camps et al. (2003)
	TADR	Medium	<4,000	*E. coli*	Transition, Transversion, Indel	2.3×10^-5^	Yi et al. (2021)
	OrthoRep	Low	<22,000	*S. cerevisiae*	Transition, Transversion, Indel (<1%)	1.0×10^-5^	Ravikumar et al. (2018)
	BacORep	Low	<15,000	* B. thuringiensis*	Transition, Transversion, Indel (<1%)	6.8×10^-7^	Tian et al. (2023)
	EcoRep	Low	<16,500	*E. coli*	Transition, Transversion, Indel (<1%)	9.13×10^-7^	Tian et al. (2024)
	T7 Repolisome	Low	-	*E. coli*	Transition, Transversion, Indel	1.7×10^-5^	Diercks et al. (2024)
	EvolvR	Low	<60	*E. coli*	Transition, Transversion, Indel	3.47×10^-6^	Halperin et al. (2018)
	yEvolvR	Low	<60	*S. cerevisiae*	Transition, Transversion, Indel	1.24×10^-6^	Tou et al. (2020)
Base editor-based mutagenesis	dCas9-BE	Low	<30	Mammalian cells	Limited transition (C→T, G→A)	Not specified	Komor et al. (2016)
	CRISPR-X	Low	<30	Mammalian cells	Limited transition (C→T, G→A)	6.25×10^-4^	Hess et al. (2016)
	CoMuTER	Low	<55,000	*S. cerevisiae*	Limited transition (C→T, G→A)	3×10^-4^	Zimmermann et al. (2023)
	HACE	Low	<1000	Mammalian cells	Limited transition (C→T, G→A)	4.26×10^-3^	Chen et al. (2024a)
	MutaT7	Low	<20,000	*E. coli*	Limited transition (C→T, G→A)	6.67×10^-6^	Moore et al. (2018)
	eMutaT7^C→T^	Low	<20,000	*E. coli*	Limited transition (C→T, G→A)	9.40×10^-5^	Park & Kim (2021)
	eMutaT7^A→G^	Low	<20,000	*E. coli*	Limited transition (A→G, T→C)	~10^-5^ (Approximation)	Mengiste et al. (2023)
	eMutaT7^transition^	Low	<20,000	*E. coli*	Transition	3.6×10^-5^	Seo et al. (2023)
	MutaT7^GDE^	Low	<20,000	*E. coli*	Transition	~10^-4^ (Approximation)	Mengiste et al. (2024)
	T7-DIVA	Low	<20,000	*E. coli*	Limited transition (C→T, G→A)	6.67×10^-5^	Álvarez et al. (2020)
	TRACE	Low	<20,000	Mammalian cells	Limited transition (C→T, G→A)	1.16×10^-4^	Chen et al. (2020)
	TRIDENT	Low	<20,000	*S. cerevisiae*	Limited transition (C→T, G→A)	1.25×10^-4^	Cravens et al. (2021)
Homologous recombination-based DNA mutagenesis	MAGE	High	<30	*E. coli*	Transition, Transversion, Indel	Not specified	Wang et al. (2009)
	eMAGE	High	<30	*S. cerevisiae*	Transition, Transversion, Indel	Not specified	Barbieri et al. (2017)
	Retron	Medium	<30	*E. coli*	Transition, Transversion, Indel	6.37×10^-7^	Simon et al. (2018)
	REGES	Medium	<300	*E. coli*	Transition, Transversion, Indel	2.4×10^-3^	Liu et al. (2023)
